# Complete Genome Sequence of *Tomato Spotted Wilt Virus*, a South Korean Isolate from Gerbera jamesonii

**DOI:** 10.1128/MRA.00608-21

**Published:** 2021-08-26

**Authors:** San Yeong Kim, Sangmin Bak, Sung Tae Kim, Eunsook Lee, Dae-Hong Lee, Jun-Hong Park, Chang-Kil Kim

**Affiliations:** a Gumi Floriculture Research Institute, Gyeongsangbuk-do Agricultural Research and Extension Services, Gumi, Republic of Korea; b School of Applied Biosciences, Kyungpook National University, Daegu, Republic of Korea; c Department of Horticultural Science, Kyungpook National University, Daegu, Republic of Korea; DOE Joint Genome Institute

## Abstract

The genome sequence of *Tomato spotted wilt orthotospovirus* (TSWV) isolated from gerbera was determined. The genome consists of L, M, and S segments containing 8,920, 4,775, and 2,970 nucleotides, respectively. BLASTn analysis showed respective identities of 99.84%, 99.71%, and 99.50% with another Korean isolate, GS, from pepper.

## ANNOUNCEMENT

*Tomato spotted wilt orthotospovirus* (TSWV) is a member of the genus *Orthotospovirus* in the family *Tospoviridae*. TSWV is widely distributed worldwide and is known to have a wide host range. TSWV infecting gerbera has been reported in Serbia and Venezuela (1, 2). In August 2019, symptoms suggestive of viral infection, such as leaf distortion and ring spot symptoms, were observed on gerbera plants in a greenhouse in Gumi, South Korea. All gerbera individuals in the greenhouse were produced through tissue culture for hydroponic cultivation experiments and expected to be virus-free. Symptoms were observed in approximately 30% (from a total of 200 plants) of the plants in the greenhouse. Fifteen symptomatic leaf samples were collected, and 9 of the 15 samples were positive by ImmunoStrip kit for TSWV (Agdia, Elkhart, IN). To confirm the presence of tomato spotted wilt virus, total RNA was extracted from all collected leaf samples using an Easy Spin total RNA extraction kit (iNtRON, Seongnam, South Korea), and reverse transcriptase PCR (RT-PCR) was performed using primers specific for TSWV (3). Total RNA extracted from TSWV-infected peppers was used as a positive control, and pure water was used as a negative control. The RT-PCR diagnosis yielded the same result as the ImmunoStrip kit. For further confirmation of TSWV presence, cDNA was synthesized using the SuperiorScript III cDNA synthesis kit (Enzynomics, Daejeon, South Korea). The random hexamer (N6) primer was used as the initiator of synthesis. PCR analysis was performed to analyze the full-length sequence.

Primers were designed in conserved regions based on the previously reported TSWV sequence (Table 1). The 5′ and 3′ termini of the L, M, and S segments were amplified using the primer J13 (4). The primer J13 contains eight nucleotides that were completely conserved in all RNAs of TSWV. As a result, 15 overlapping fragments were amplified and cloned using an All in One cloning kit (BioFact, Daejeon, South Korea). Sequencing reactions were performed using the BigDye Terminator v3.1 cycle sequencing kit (Thermo Fisher Scientific, Waltham, MA). PCR was performed using the DNA Engine Tetrad 2 Peltier thermal cycler (Bio-Rad, Hercules, CA). After the reaction was completed, deoxynucleoside triphosphates (dNTPs) and reactants not participating in the reaction were removed by the method recommended by the manufacturer and then loaded into an ABI 3730xl DNA analyzer (Thermo Fisher Scientific). All sequencing reactions were commissioned to Macrogen (Seoul, South Korea). The assembly of each of the obtained fragments and removal of nonconserved regions on the J13 primer were performed using UGENE software v37.1 (Unipro, Novosibirsk, Russia). The complete genome sequence included L, M, and S segments, which contain 8,920, 4,775, and 2,970 nucleotides, respectively (GenBank accession numbers MW048592, MW048591, and MW048590). Six open reading frames (ORFs) were predicted in the complete genome sequence using NCBI ORF Finder (https://www.ncbi.nlm.nih.gov/orffinder/). The tool was run using default parameters. The GC content of each segment was 33.58%, 35.35%, and 34.41%, respectively. Each sequence was compared to the NCBI GenBank database using the BLASTn option from BLAST+ v2.11.0 as the default parameter (https://blast.ncbi.nlm.nih.gov/Blast.cgi). The results of the analysis showed identities of 99.84%, 99.71%, and 99.50% with another Korean isolate, GS (MF159043, MF159055, and MF159067), from pepper. The sequence of the nucleocapsid (N) gene showed 96.78% identity with the Venezuelan isolate (KF146703), extracted from gerbera. Furthermore, *Frankliniella occidentalis* (western flower thrips), known as the major vector of TSWV, was found inside the greenhouse (5). This suggests that TSWV may have been introduced into the greenhouse from an external source. Given the wide host range, TSWV may have been effectively transmitted by vectors without genetic alterations.

**TABLE 1:** List of primers used for cloning and sequencing of *Tomato spotted wilt virus* isolate Gumi

Target RNA	Primer name^*a*^	Sequence (5′ to 3′)	Position	Expected size (bp)
L segment	J13	CCCGGATCC**AGAGCAAT**		1,512
	TSWV-LF-R1503	GTTGTAATCTACCTCTGAGCTC	1503–1482	
	TSWV-LF-F1284	GAAACACCTTGAGAAGATCATGC	1284–1306	1,450
	TSWV-LF-R2733	CATCCTGCTGAGCTTTGTC	2733–2715	
	TSWV-LF-F2508	GAGACTCAACCAGGTGAGG	2508–2526	917
	TSWV-LF-R3424	CTATATCTTGGATCTTTGCATTC	3424–3402	
	TSWV-LF-F3271	GGTTTGATGAGATCAGAAATAG	3271–3292	1,382
	TSWV-LF-R4652	GGGTTCAAAGTTATGCAAAAGC	4652–4631	
	TSWV-LF-F4551	GGTCGATAAAATGCTAACAGAC	4551–4572	1,581
	TSWV-LF-R6131	CATTCATTAGAATTGCTGAAAGCC	6131–6111	
	TSWV-LF-F6033	CACGTTTGGAACAGGTTTAATC	6033–6054	1,325
	TSWV-LF-R7357	CTAAGCAGGTATCCCGGATG	7357–7338	
	TSWV-LF-F7260	CTTTGGAAACTTATCACAGCAG	7260–7281	1,328
	TSWV-LF-R8587	CATCTGTTTCAACAACCTCATC	8587–8566	
	TSWV-LF-F8146	GATATAGAGACATTGTTGCGG	8146–8166	773
	J13	CCCGGATCC**AGAGCAAT**		
M segment	J13	CCCGGATCC**AGAGCAAT**		1,414
	TSWV-MF-R1405	CTAGATCCAAGATAGAGGATG	1405–1385	
	TSWV-MF-F1271	CAAATTTAGCCTGTGACAAGC	1271–1291	1,592
	TSWV-MF-R2862	GCCTAGACAATCATTGATCTTTG	2862–2840	
	TSWV-MF-F2545	CATCCCCAATAAGAAGTTGG	2545–2564	1,593
	TSWV-MF-R4137	CTTCATTTCAGAAAGCCTGAC	4137–4117	
	TSWV-MF-F3838	GATTTGTCACCGCACAAGAG	3838–3857	959
	J13	CCCGGATCC**AGAGCAAT**		
S segment	J13	CCCGGATCC**AGAGCAAT**		1,451
	TSWV-SF-R1442	TGATCCCGCTTAAATCAAGC	1442–1423	
	TSWV-SF-F1149	GCAACAACTTGCAAGAAGATG	1149–1169	1,406
	TSWV-SF-R2554	CATGACCTTCAGAAGGCTTG	2554–2535	
	TSWV-SF-F2326	CAAGACAACACTGATCATCTC	2326–2346	641
	J13	CCCGGATCC**AGAGCAAT**		

a The underlined J13 primer contains nucleotides (bold) known to be completely conserved at the tospovirus RNA terminus (4). The J13 primer was used at the 5′ and 3′ termini of each RNA segment.

### Data availability.

The complete genome sequences of the L, M, and S segments of TSWV isolate Gumi were deposited in GenBank under the accession numbers MW048592, MW048591, and MW048590, respectively.
